# Novel Senescent Regulatory T-Cell Subset with Impaired Suppressive Function in Rheumatoid Arthritis

**DOI:** 10.3389/fimmu.2017.00300

**Published:** 2017-03-20

**Authors:** Johannes Fessler, Andrea Raicht, Rusmir Husic, Anja Ficjan, Christine Schwarz, Christina Duftner, Wolfgang Schwinger, Winfried B. Graninger, Martin H. Stradner, Christian Dejaco

**Affiliations:** ^1^Department of Rheumatology and Immunology, Medical University of Graz, Graz, Austria; ^2^Department of Pediatric Hemato-Oncology, Medical University of Graz, Graz, Austria; ^3^Department of Internal Medicine VI, Innsbruck Medical University, Innsbruck, Austria; ^4^Rheumatology Service, South Tyrolian Health Trust, Hospital Bruneck, Bruneck, Italy

**Keywords:** regulatory T cells, rheumatoid arthritis, immunosenescence, aging, premature, autoimmunity

## Abstract

**Objective:**

Premature senescence of lymphocytes is a hallmark of inflammatory rheumatic diseases such as rheumatoid arthritis (RA). Early T-cell aging affects conventional T-cells but is presumably not limited to this cell population; rather it might also occur in the regulatory T-cells (Tregs) compartment. In RA, Tregs fail to halt aberrant immune reactions and disease progression. Whether this is associated with early Treg senescence leading to phenotypic and functional changes of this subset is elusive so far.

**Methods:**

Eighty-four RA patients and 75 healthy controls were prospectively enrolled into the study. Flow cytometry, magnetic-associated cell sorting, and cell culture experiments were performed for phenotypic and functional analyses of Treg subsets. T-cell receptor excision circle (TREC) levels and telomere lengths were determined using RT-PCR.

**Results:**

In this paper, we describe the novel CD4^+^FoxP3^+^CD28^−^ T-cell subset (CD28^−^ Treg-like cells) in RA patients revealing features of both Tregs and senescent T-cells: Treg surface/intracellular markers such as CD25, CTLA-4, and PD-1 as well as FOXP3 were all expressed by CD28^−^ Treg-like cells, and they yielded signs of premature senescence including reduced TREC levels and an accumulation of γH2AX. CD28^−^ Treg-like could be generated *in vitro* by stimulation of (CD28^+^) Tregs with TNF-α. CD28^−^ Treg-like cells insufficiently suppressed the proliferation of effector T-cells and yielded a pro-inflammatory cytokine profile.

**Conclusion:**

In conclusion, we describe a novel T-cell subset with features of Tregs and senescent non-Tregs. These cells may be linked to an aberrant balance between regulatory and effector functions in RA.

## Introduction

Regulatory T-cells (Tregs) were identified as sentinels of the immune response keeping aberrant immune reactions in track and preventing autoimmunity. The current understanding of the pathogenesis of rheumatoid arthritis (RA) is that this equilibrium is disturbed, in part due to quantitative and/or qualitative defects of Tregs [reviewed in Ref. (1)]. Currently available data on this topic, however, are contradictory in part: Lawson et al., for example, observed a reduced prevalence of circulating CD4^+^CD25^high^ Tregs in patients with early active RA (2), whereas others were unable to reproduce this finding (3, 4). Ehrenstein et al. reported a defect of Tregs in the suppression of pro-inflammatory cytokine production (5), and Nie et al. showed a reduced suppression of proliferating effector cells by Tregs that was TNF dependent (6). Other groups, however, reported that the suppressive function of Tregs in RA is normal (3, 4, 7).

These discrepancies are potentially linked to the fact that Tregs are not a single cell population but rather comprise developmentally and functionally distinct subsets that may vary among RA populations. Similar to non-Tregs are educated in the thymus and are then released in a “naïve-like” CD25^+^CD45RA^+^ phenotype into periphery (8, 9). Upon antigen contact, Tregs differentiate into a “memory-like” CD25^hi^CD45RO^+^ phenotype (10). Memory-like Tregs have a lower proliferative capacity and different homing behavior compared to naive-like Tregs and yield shorter telomeres as well as a lower content of T-cell receptor excision circles, an extrachromosomal DNA byproducts of T-cell receptor (TCR) rearrangement that is diluted by peripheral T-cell division, indicating a longer replicative history.

During aging, the thymus undergoes progressive involution leading to an elevated homeostatic pressure on peripheral T-cells (11, 12). Proliferation of peripheral T-cells compensates for dwindling thymic output until telomeres are contracted to levels known as the “Hayflick limit.” At this stage, non-Tregs undergo senescence-associated phenotypical and functional changes such as downregulation of CD28. Due to the fact that Tregs display even shorter telomeres than non-Tregs, it is conceivable that Tregs proliferating in periphery reach the “Hayflick limit” even earlier (13). Impaired Treg homeostasis and function might then result in an increased risk of immune-mediated disorders.

In RA, senescence of non-Treg including the accumulation of CD28^−^CD4^+^ T-cells has been observed even in young individuals (14, 15). CD28^−^ T-cells from RA patients are pro-inflammatory and cytotoxic, and a high prevalence of this cellular subset has been associated with higher disease severity and cardiovascular events (14, 16–18). Whether in RA, premature senescence including the loss of CD28 molecule also affects Tregs has not been investigated so far. From a mouse model with a conditional CD28 knockout in Tregs we know that the loss of this co-stimulatory molecule severely compromises Treg function leading to the development of autoimmunity (19).

This study aims at the identification and characterization of CD4^+^CD28^−^FoxP3^+^ T-cells in patients with RA.

## Patients and Methods

### Study Population

This was a prospective study of consecutive patients with a final diagnosis of RA based on the 2010 ACR/EULAR criteria (20) as well as age- and sex-matched healthy individuals (HC). Detailed medical history including disease duration, prior, and current treatments was obtained from each patient. Each patient also underwent full clinical assessment with determination of disease activity according to the simplified disease activity index (SDAI) (21) and the disease activity score 28 (22). Whenever available, synovial fluid samples were obtained from patients undergoing routine joint aspiration.

### Peripheral Blood Mononuclear Cells (PBMCs) and Cell Culture

Peripheral venous blood or synovial fluid was drawn from each individual, and PBMCs were isolated by Histopaque density gradient centrifugation. The total cell number was determined by a Beckmann Coulter. Cells were cultured at 1 × 10^6^ cells/ml in RPMI 1640 containing 10% fetal calf serum, 2 mM l-glutamine, 100 U/ml penicillin, and 100 μg/ml streptomycin in the presence of 20 U/ml human recombinant IL-2 (SIGMA, Vienna, Austria) and initial stimulation with 10 μg/ml plate-bound anti-CD3 Ab.

### Flow Cytometry

Surface and intracellular staining of freshly isolated PBMCs was performed using appropriate combinations of antibodies for detection of CD3, CD4, CD28, CD25, CD127, FoxP3, CTLA-4, IL-10, TNF-α, Th1-type cytokines IL-2 and IFN-γ, Th2-type cytokine IL-4 and Th17-type cytokine IL-17 (all Becton Dickinson, San Diego, CA, USA), and γH2AX (Cell Signaling, Danvers, MA, USA). Surface staining for 20 min was followed by permeabilization for 30 min and intracellular staining for 30 min according to a routine protocol. Golgi transport was inhibited by brefeldin A (10 ng/ml) or monensin (10 ng/ml) 4 h prior to cytokine staining. Appropriate isotype controls were used. Stained cells were analyzed on a FACS Canto II (Becton Dickinson). Data are analyzed with DIVA software and FlowJo.

### Isolation of T-Cell Subsets

For functional assays, CD4^+^ T-cells were isolated by positive selection of PBMCs labeled with magnetic-bead conjugated antihuman CD4 mAbs using MACS MultiSort Kit and autoMACSPro according to manufacturer’s instructions (Miltenyi). Purified CD4^+^ T-cells were then separated into the CD28^+^CD25^+^CD127^dim^ (conventional regulatory), CD28^+^CD25^−^ (conventional non-regulatory), and CD28^−^CD25^+^CD127^dim^ (senescent Treg-like) fractions by another sorting step using FACS technology (FACS Aria). In order to obtain sufficient numbers of RA Tregs, pre-enrichment of PBMCs was necessary and resulted in upregulation of CD25. Therefore, isolation of Treg subsets was based on CD25 positivity as well as absence of CD127. For CD28 downregulation experiments, Tregs were isolated using CD4^+^CD25^+^CD127^dim/−^ Reg. T Cell isolation Kit II (Miltenyi) and autoMACSPro. For validation, flow cytometry was then performed to determine purity (>90%) of selected cells.

### Determination of TCR Diversity

RNA from enriched subsets was extracted using RNeasy Protect Mini Kit (Qiagen, Germantown, MD, USA) according to the manufacturer instructions. RNA was used for reverse transcription with First Strand cDNA Synthesis Kit for RT-PCR AMV (Roche) according to the manufacturer regulations. cDNA was diluted 1:5 for PCR using AmpliTaq Gold™ DNA Polymerase (Applied Biosystems), 1× PCR Gold Buffer (Applied Biosystems), 2.5mM MgCl_2_ (Applied Biosystems), 0.4 mM dNTP Polymerization Mix (GE Healthcare), 0.5 μM TCR C β 5′FAM labeled primer (Ingenetix, Vienna, Austria), and 0.5 μM unlabeled TCR V β primers (Ingenetix) according to Hingorani et al. (23). Primer sequences are listed in Table S2 in Supplementary Material. Cycle conditions were a denaturation step at 94°C for 6 min, 35 cycles of 94°C for 1 min, 59°C for 1 min, and 72°C for 1 min, a final annealing step at 72°C for 7 min. After amplification, the PCR product was supplemented with GeneSCan™-500 TAMRA™ Size Standard (Applied Biosystems) and HI-DI Formamide (Applied Biosystems). Electrophoresis was performed with ABI Prism 310 Genetic Analyzer (Applied Biosystems), and 310 Data Collection Software. Analysis was done by GeneScan^®^ Software (Applied Biosystems). Calculations included peak count (Complexity score) and single peak area in percent of whole peak area.

#### Senescence-Associated β-Galactosidase Assay

Freshly isolated PBMCs were treated with the “Quantitative Cellular Senescence Assay Kit” (Cell Biolabs) according to the manufacturer’s instructions. Afterward, a surface and intracellular staining of cells was performed as described above.

### Determination of Telomere Length

DNA from enriched subsets was extracted using QIAamp DNA Blood Mini Kit (Qiagen, Germantown, MD, USA) according to the manufacturer instructions. Telomere lengths were measured using DNA from PBMCs as well as T-cell subsets by quantitative real-time PCR analysis and LightCycler FastStart DNA Master SYBR Green I (Roche, Vienna, Austria) as previously described (24).

### Suppression Assay

CD4^+^ T-cells were isolated always from the same healthy donor (JF) since RA effector cells were reported to be resistant to Treg-mediated suppression (5). Cells were incubated with CellTrace Violet (Life Technologies) and cultured in the presence or absence of CD28^+^CD25^+^CD127^dim^ or CD28^−^CD25^+^CD127^dim^ RA T-cells at a 1:1 ratio. Autologous CD28^+^CD25^+^CD127^dim^ served as a control. Cells were stimulated by adding 10 μg/ml anti-CD3 Ab and incubated at 37°C for 3 days and analyzed by flow cytometry. To elucidate the impact of TNF-α and IFN-γ on the suppressive potential of different subsets, we added neutralizing antibodies (R&D systems) at 10 μg/ml. A normal goat IgG control was also added at the start of culture accounting for any non-specific changes.

#### Proliferation Assay

For all experiments, freshly purified PBMCs were resuspended in PBS at 5–10 × 10^6^ cells/ml and incubated with CFSE (1 μM) for 7 min at 37°C. Cells were washed three times and resuspended in culture medium. Cells were stimulated with plate-bound anti-CD3 (10 μg/ml, 18 h) or PHA (1 μg/ml) and cultured for 72 h. Afterward cells were stained as described in Section “Flow Cytometry.”

### Apoptosis Assay

Freshly isolated PBMCs were stimulated with plate-bound anti-CD3 (10 μg/ml) or PHA (1 μg/ml) and cultured for 18 h. Afterward cells were harvested and stained for Annexin V and viability dye (ebioscience, San Diego, CA, USA) as well as surface markers according to the manufacturer’s protocol. Annexin V^+^ cells represent apoptotic cells, viability dye^+^ cells represent necrotic cells, and double positive cells represent late apoptotic cells.

#### CD28 Downregulation Experiments

CD4^+^CD25^+^CD127^dim/−^ Tregs were isolated using AutoMACS (Miltenyi, Bergisch Gladbach, Germany) according to the manufacturer’s instructions. Subsequently, cells were cultured as described (25): in brief, 1 × 10^5^ cells/ml were cultured in a 96-well plate with culture media and anti-CD3/CD28 MoAb-coated microbeads (Life Sciences, Waltham, MA, USA) at a 4:1 bead-to-cell ratio and were stimulated with 200 U/ml IL-2, with or without 100 ng/ml TNF-α (SIGMA) or IL-15 (Life Sciences). After 6 days of cultivation, the cells were harvested, washed, and then cultured in the presence of 20 U/ml IL-2 for two more days. Thereafter, the expanded Tregs were restimulated for another 6 days with anti-CD3/CD28 MoAb-coated microbeads at a bead-to-cell ratio of 2:1 and 200 U/ml IL-2. Cells were analyzed after each expansion phase.

### Statistical Analysis

All statistical analyses were performed using the SPSS program, version 23 (Chicago, IL, USA). In case of a normal distribution (tested with the Kolmogorov–Smirnov test) of the continuous variables, the mean and SD are shown, and we used the two-sided Student’s *t*-test (comparison of two groups) or ANOVA (comparison of three or more groups) for comparisons. In case of a non-parametric distribution, the median and range are shown, and we used the Mann–Whitney *U* and the Kruskal–Wallis tests to assess differences between groups. Correlation between variables was evaluated by the Spearman’s rank correlation coefficient. Paired data were compared with the Wilcoxon test.

### Study Approval

This study was approved by the Institutional Review Board of the Medical University Graz, and written informed consent was obtained from each individual prior to inclusion in the study.

## Results

### CD4^+^CD28^−^FoxP3^+^ T-Cells Have a Treg-Like Phenotype and Are Prevalent in RA

We know that in RA, (1) a proportion of T-cells lack the co-stimulatory molecule CD28, (2) the loss of CD28 reflects early T-cell senescence and is partially caused by pro-inflammatory stimuli, and (3) Tregs undergo a similar development to non-Tregs from a naïve-like to a memory-like status. We therefore investigated whether expression of CD28 is reduced on FoxP3^+^ T-cells (which is the most specific marker for Tregs) from RA patients.

**Figure 1:**
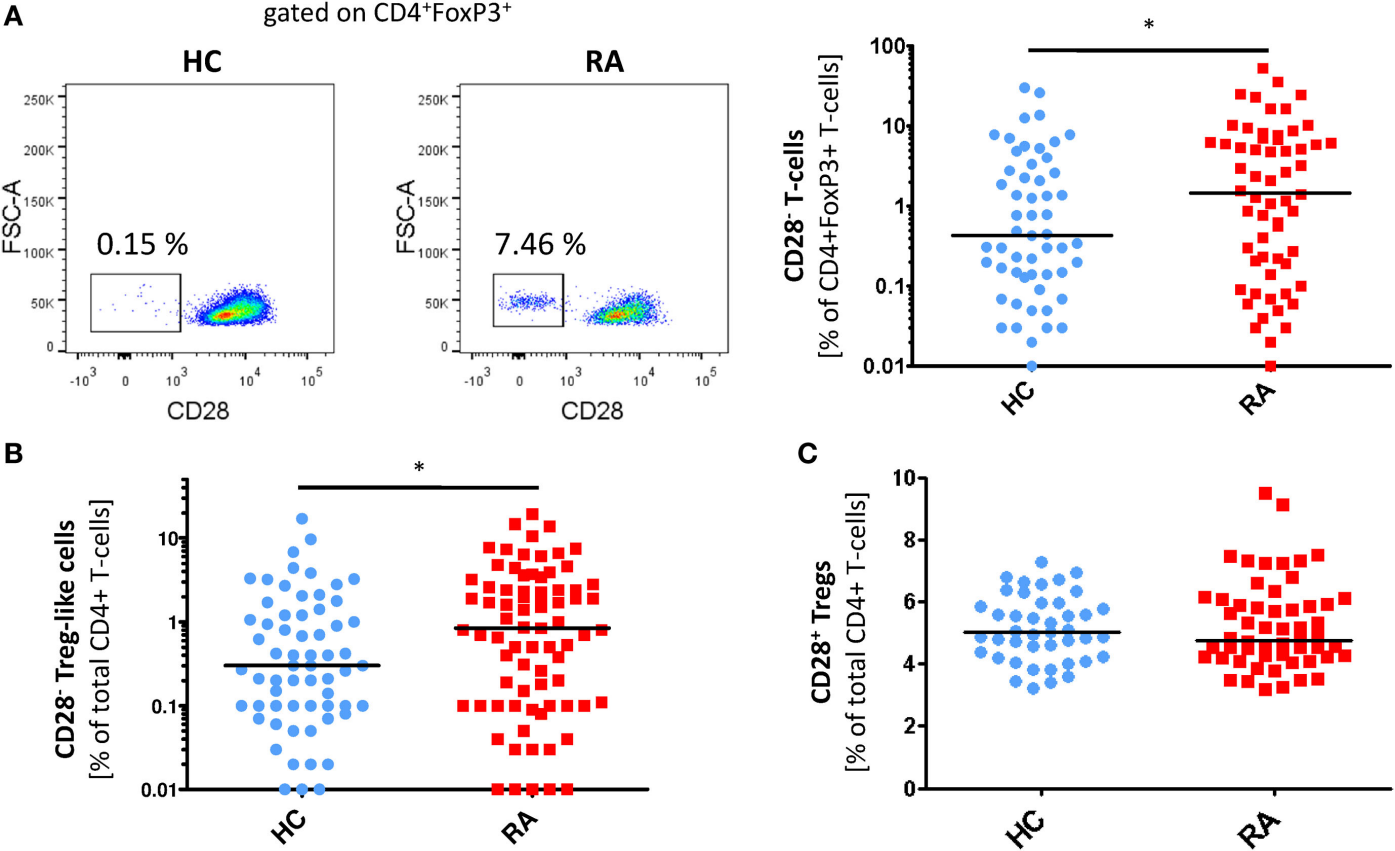
**CD28^−^ regulatory T-cells (Tregs)-like cells are enriched in patients with rheumatoid arthritis (RA)**. Graphs show **(A)** representative dot plots of CD28^−^ Treg-like cells (gated on CD4^+^FoxP3^+^ T-cells) of patients with RA (*n* = 84) and HC (*n* = 75) and statistical analysis as well as **(B)** prevalences of CD28^−^ Treg-like cells and **(C)** CD28^+^ Tregs of healthy controls (HC, blue) and RA patients (red); Mann–Whitney *U* test and Student’s *t*-test, respectively, were used to assess differences between groups. *p ≤ 0.05.

**Figure 2:**
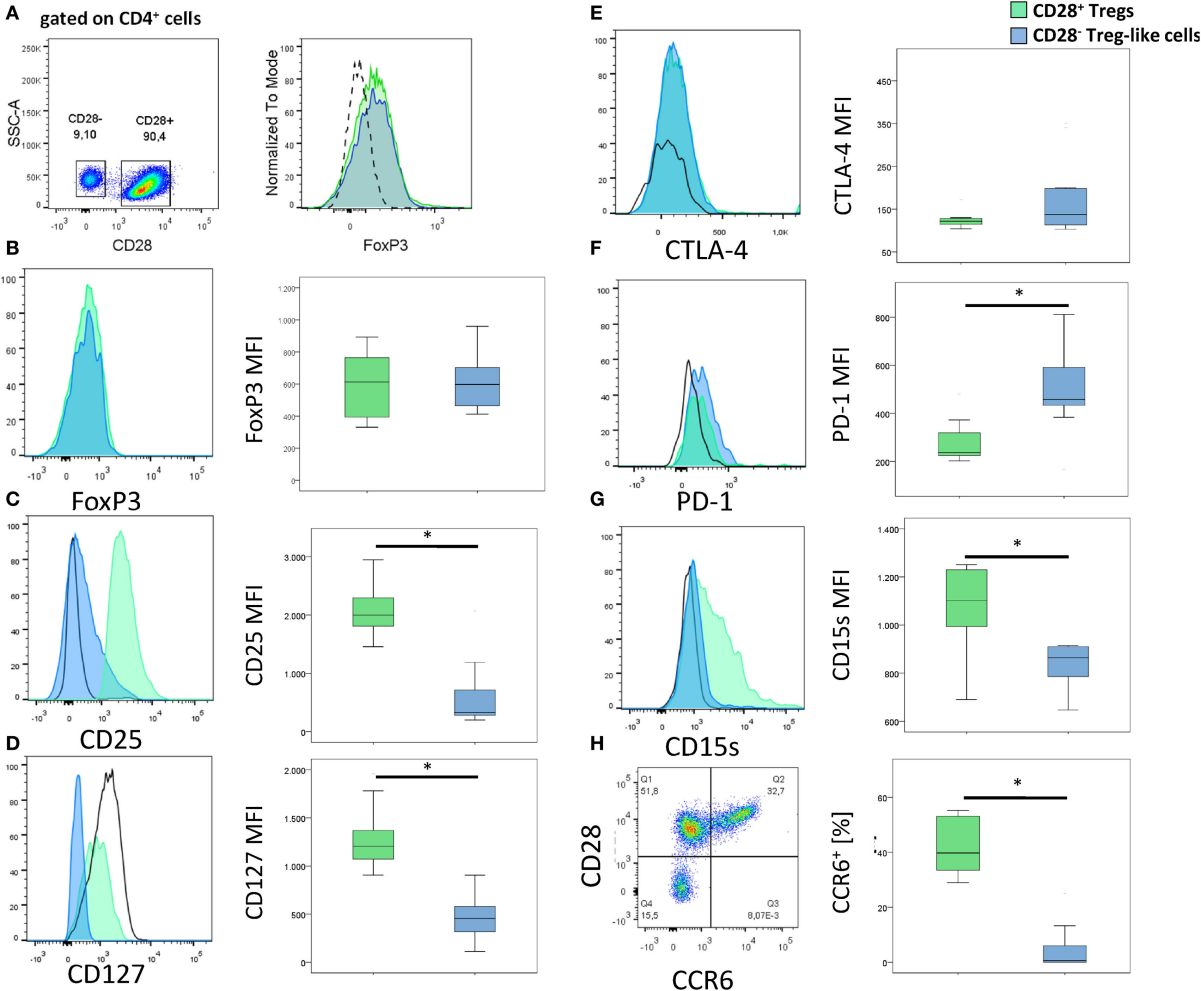
**Surface markers of CD28^−^ regulatory T-cells (Tregs)-like cells and CD28^+^ Tregs**. **(A)** Gating strategy for the identification of CD28^−^ Treg-like cells (gated on CD4^+^ cells). **(B–H)** Graphs show representative histograms or dot plots and box plots of 10 rheumatoid arthritis patients of **(B)** FoxP3, **(C)** CD25, **(D)** CD127, **(E)** CTLA-4, **(F)** PD-1, **(G)** CD15s, and **(H)** CCR6 expression of CD28^+^ Tregs (green), CD28^−^ Treg-like cells (blue), and conventional (FoxP3^−^) T-cells (black solid line). Isotype control expression levels are indicated by dashed lines. Mann–Whitney *U* test was used to assess differences between groups. *p < 0.05.

In RA patients but not in controls, we observed a FoxP3^+^ T-cell subset lacking the expression of CD28. The prevalence of circulating CD4^+^CD28^−^FoxP3^+^ T-cells was higher in RA patients compared to healthy individuals [0.7% of total CD4^+^ (range 0–19.2) vs. 0.2% (0–17); *p* = 0.029; Figures 1A,B], whereas the frequency of CD4^+^CD28^+^FoxP3^+^CD25^+^ Tregs was equal in both groups [5% of total CD4^+^ (3.2–7.3) vs. 4.9% (3.2–9.5); *p* = 0.988; Figure 1C]. The intensity of FoxP3 expression was similar in CD28^−^ and CD28^+^ subsets (MFI: 601.8 ± 200.4 vs. 634.8 ± 196.5, *p* > 0.05, Figures 2A,B). In synovial fluid samples from RA patients, we noted that 3.8% (1.6–18.8) of CD4^+^FoxP3^+^ T-cells were negative for CD28 (*n* = 8, data not shown).

Alongside FoxP3, CD25 and a low expression of CD127 are characteristic for Tregs (26, 27). CD4^+^ CD28^−^FoxP3^+^ T-cells were positive for CD25 and expressed low levels of CD127; both markers, however, were reduced relative to CD4^+^CD28^+^FoxP3^+^ Tregs: CD25, MFI: 328 (199–2,068) vs. 1,998 (1,456–2,946); *p* < 0.001; Figure 2C and CD127, MFI: 454 (111–905) vs. 1,206 (906–1,961); *p* < 0.001; Figure 2D. CTLA-4, PD-1, and CD15s are other typical markers of Tregs, all of them were found on CD4^+^CD28^−^FoxP3^+^: CTLA-4 expression was similar in CD4^+^CD28^−^FoxP3^+^ T-cells and CD4^+^CD28^+^FoxP3^+^ Tregs [MFI: 123.5 (103–350) vs. 122 (104–648); *p* = 0.499; Figure 2E], whereas PD-1 was higher [MFI: 449.5 (167–811) vs. 258.5 (221–480); *p* = 0.017; Figure 2F] and CD15s lower [MFI: 864.5 (647–914) vs. 1,102.5 (690–1,251); *p* = 0.028; Figure 2G] in the former compared to the latter cell population. CCR6, another molecule normally expressed by Tregs was almost absent on CD4^+^CD28^−^FoxP3^+^T-cells [0.2% (0–7.5) of CD4^+^CD28^−^FoxP3^+^ T-cells were positive for CCR6^+^ vs. 39.8% (28.9–55.2) of CD28^+^ Tregs; *p* = 0.012, Figure 2H] (28).

Collectively, these analyses suggest that CD4^+^CD28^−^FoxP3^+^ T-cells have a Treg-like phenotype.

### CD28^−^ Treg-Like Cells Underwent Cellular Senescence

To investigate whether the novel CD28^−^ Treg-like subset revealed signs of cellular senescence in addition to the loss of CD28, we undertook the following experiments: first, we tested the accumulation of γH2AX foci, which represent repair-proof double-strand breaks in DNA (29). Second, we performed TCR spectratyping because TCR diversity is known to diminish along with T-cell aging (30). Third, we tested the accumulation of senescence-associated β-galactosidase (SABG) another known biomarker of cellular senescence (31). Fourth, we measured the telomere length given that a shrinkage of telomeres is considered to indicate replicative senescence (32).

**Figure 3:**
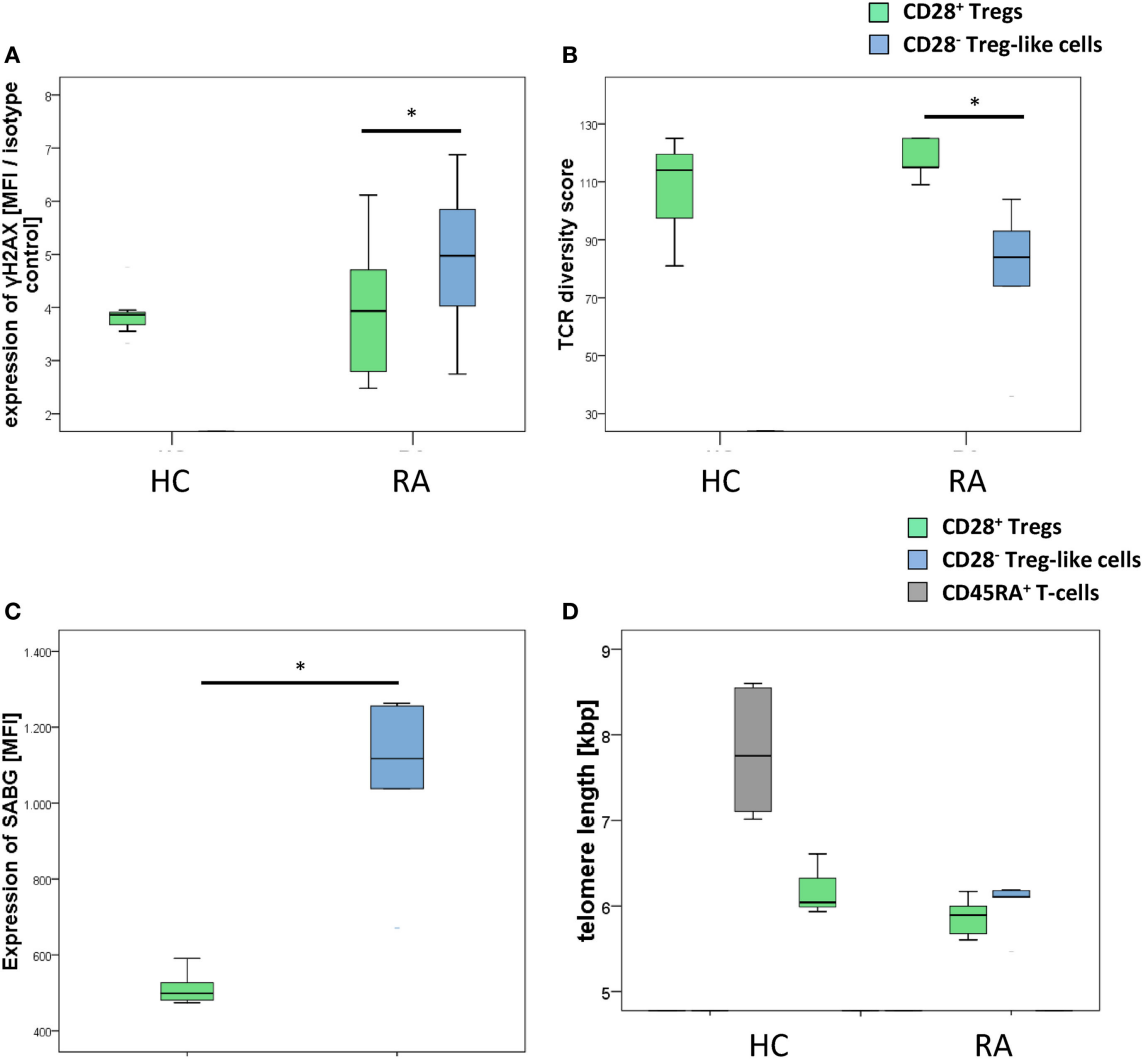
**CD28^−^ regulatory T-cells (Tregs)-like cells show evidence of advanced replicative senescence**. Graphs show **(A)** expression of γH2AX, **(B)** T-cell receptor diversity, **(C)** expression of senescence-associated β-galactosidase (SABG), and **(D)** telomere lengths of CD28^+^ Tregs (green), CD28^−^ Treg-like cells (blue), and naive CD45RA^+^ T-cells (gray). Mann–Whitney *U* test was used to assess differences between groups. *p < 0.05; *n* = 5.

As depicted in Figure 3A, CD28^−^ Treg-like cells showed higher γH2AX mean fluorescence intensity than CD28^+^ Tregs [MFI: 6,422 (2,952–258,589) vs. 4,875 (2,875–7,743), *p* = 0.046]. Similarly, TCR diversity was reduced in CD28^−^ Treg-like cells [84 (36–104)] compared to their CD28^+^ counterparts [115 (109–125); *p* = 0.037; Figure 3B]. SABG expression, moreover, was likewise enhanced in CD28^−^ Treg-like cells [MFI: 1,117 (671–1,263) vs. 499 (474–591); *p* = 0.043; Figure 3C].

Interestingly, telomere length was similar in CD28^−^ Treg-like cells [6.11 kbp (5.46–6.19)] and CD28^+^ Tregs [5.89 (5.6–6.17), *p* = 0.373, Figure 3D]. From previous studies we know, however, that the telomere length of Tregs is reduced already (compared to non-Tregs), and a further shrinkage of telomeres is thus unlikely to occur (13).

### Association of CD28^−^ Treg-Like Cells with Clinical Parameters of RA

Clinical characteristics of RA patients and controls are depicted in Table S1 in Supplementary Material. Two (3.3%), 8 (13.1), and 32 (52.5) out of the 61 RA patients with available SDAI values had high, moderate, or low disease activity, respectively; 19 (31.1) patients were in clinical remission (33). Five (6%) RA patients had early disease (≤2 years’ duration).

Frequencies of CD28^−^ Treg-like cells (but not those of CD28^+^ Tregs) correlated with age in RA patients and controls (corr_coeff_ = 0.416, *p* < 0.001 and corr_coeff_ = 0.557, *p* < 0.001, respectively; Figure S1A in Supplementary Material). Neither the prevalence of CD28^−^ Treg-like cells nor those of CD28^+^ Tregs was linked with disease duration, acute phase reactants, clinical variables, or treatment (Figures S1B,C in Supplementary Material and data not shown).

### CD28^−^ Treg-Like Cells Can Be Generated *In Vitro*

**Figure 4:**
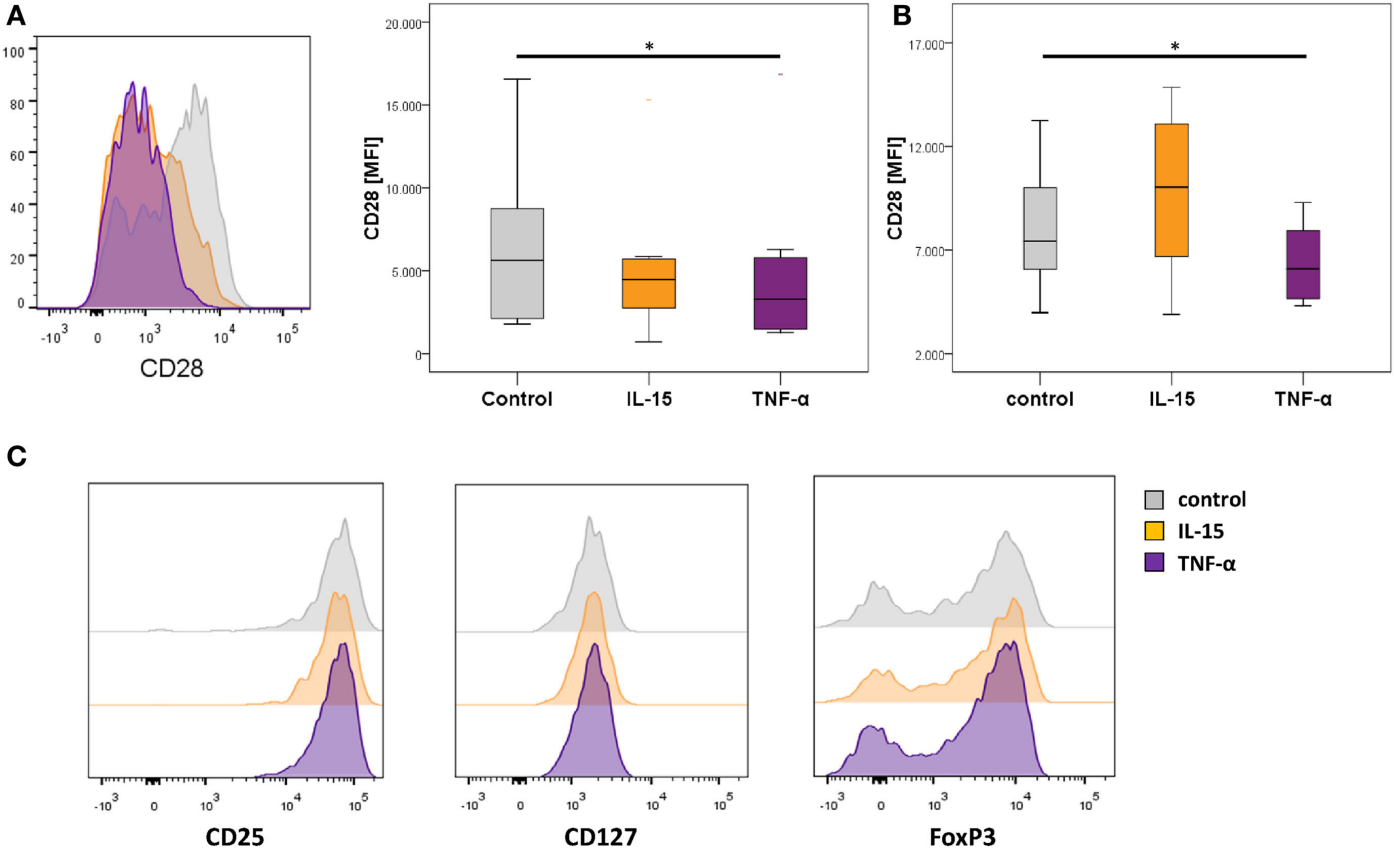
***In vitro* downregulation of CD28 in regulatory T-cells (Tregs) in the presence of TNF-α**. Graphs show **(A)** representative histograms showing CD28 expression of control Tregs (gray), following IL-15 stimulation (orange), and following TNF-α stimulation (violet), and box plots show median expression of CD28 (MFI) in Tregs of eight healthy individuals after the first expansion phase, **(B)** the second expansion phase, respectively; and **(C)** representative histograms of CD25, CD127, and FoxP3 expression. Mann–Whitney *U* test was used to assess differences between groups. *p < 0.05.

Previous studies reported that the downregulation of CD28 on T-cells is driven by pro-inflammatory signals including TNF-α or IL-15, which are also highly expressed in RA patients (34, 35). To test if these agents mediate the generation of CD28^−^ Treg-like cells *in vitro*, we isolated CD4^+^CD25^high^CD127^dim/−^ Tregs from healthy individuals and cultured them in the presence or absence of IL-15 or TNF-α. We observed a significant decrease of CD28 on Tregs stimulated with TNF-α [3,295 (1,293–16,853)] compared to mock stimulation [5,628.5 (1,782–16,559); *p* = 0.025, Figure 4A]. A decrease of CD28 was also observed following incubation with IL-15. This difference, however, did not reach statistical significance [4,483 (713–15,309); *p* = 0.138]. Moreover, the reduced expression of CD28 was robust following elongated stimulation with TNF-α but not IL-15 (Figure 4B). Expression of CD25, CD127, and FoxP3 was not different following either way of stimulation and remained in Treg-specific expression levels (Figure 4C).

### CD28^−^ Treg-Like Cells Produce High Levels of Pro- and Anti-inflammatory Cytokines

Next, we examined the cytokine profile of CD28^−^ Treg-like cells from RA patients. CD28^+^ Tregs are known to produce high levels of IL-10, which is one way how they inhibit effector cells (36). CD28^−^ non-Tregs acquire a senescence-associated secretory phenotype, which is characterized by the predominate release of Th1 cytokines such as TNF-α and IFN-γ (37).

CD28^−^ Treg-like cells yielded a more pronounced production of IL-10 than CD28^+^ Tregs following stimulation with anti-CD3 [4% (1.5–13.1) of CD28^−^ Treg-like cells vs. 0.55% (0.3–1.6) of CD28^+^ Tregs were positive for IL-10; *p* = 0.005, Table 1]. At the same time, however, cytokines characteristic for Th1 (IL-2, TNF-α, and IFN-γ), Th2 (IL-4), and Th17 lineages (IL-17) were also more frequently produced by CD28^−^ Treg-like cells compared to CD28^+^ Tregs (all *p* = 0.005). The largest difference was observed concerning the production of IL-4 (14-fold).

**Table 1:** **Cytokine production of CD28^+^ regulatory T-cells (Tregs) as well as CD28^−^ Treg-like cells following stimulation *in vitro***.

	% of CD28^−^ Treg-like cells	% of CD28^+^ Tregs	*p*-Value^a^	% of conv.CD28^−^ T-cells	*p*-Value^b^
IL-2	15.45 (5.1–31.1)	4.55 (1.1–12)	0.005	8.8 (3.6–35.6)	0.114
IL-4	4.3 (1.2–7.9)	0.3 (0.1–0.8)	0.005	3.75 (1.3–11.5)	0.005
IL-10	4 (1.5–13.1)	0.55 (0.3–1.6)	0.005	4.15 (0.5–13.4)	0.594
IL-17	4.9 (1.5–14.6)	0.7 (0.2–1.7)	0.005	4.05 (1.6–9.7)	0.203
TNF-α	18 (5.7–34.9)	11.5 (4.8–18.3)	0.005	13.6 (3.4–37)	0.074
IFN-γ	16.6 (4.3–40.4)	6.8 (2.4–16.1)	0.005	10.05 (2.8–36.4)	0.022

*^a^CD28^−^ Treg-like cells vs. CD28^+^ Tregs*.

*^b^CD28^−^ Treg-like cells vs. conv.CD28^−^ T-cells*.

Conventional senescent CD4^+^ T-cells produced lower amounts of IL-4 and IFN-γ than CD28^−^ Treg-like cells, however, no difference was found regarding the production of IL-10. For details see Table 1.

### CD28^−^ Treg-Like Cells Are Less Suppressive than CD28^+^ Tregs

**Figure 5:**
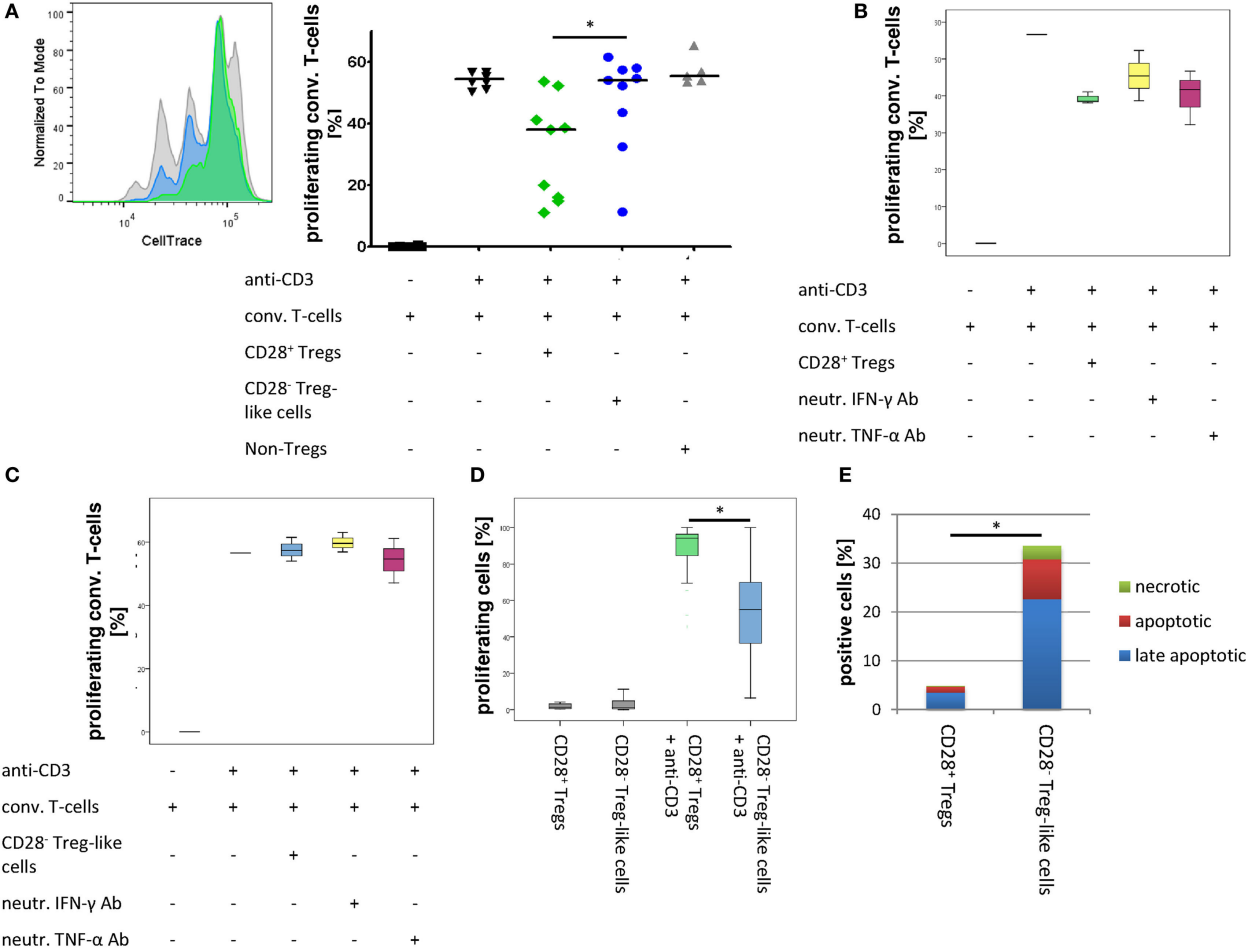
**CD28^−^ regulatory T-cells (Tregs)-like cells show reduced suppressive capacity**. Graphs show **(A)** representative histograms and box plots of *in vitro* suppression assays with CD28^+^ Tregs (green), CD28^−^ Treg-like cells (blue), as well as conventional T-cells (gray) of nine rheumatoid arthritis patients, **(B)** box plots of *in vitro* suppression assays with CD28^+^ Tregs (green) as well as **(C)** CD28^−^ Treg-like cells (blue) in the presence of neutralizing ab to IFN-γ (yellow) or TNF-α (pink); **(D)** proliferative potential of CD28^+^ Tregs (green) and CD28^−^ Treg-like cells (blue) following stimulation with anti-CD3; and **(E)** apoptotic (green), late apoptotic (blue), as well as necrotic (red) cells. Mann–Whitney *U* test was used to assess differences between groups. *p < 0.05.

The suppression of effector T-cells is a defining feature of regulatory cells. Tregs from mice with a conditional knockout for CD28 are non-suppressive, and in RA patients, Treg function has been reported (at least by some authors) to be defective (5, 19). Therefore, we studied the ability of CD28^−^ Treg-like cells to suppress the proliferation of non-Tregs. As depicted in Figure 5A, the suppressive function of CD28^−^ Treg-like cells from RA patients was compromised compared to that of CD28^+^ Tregs [median reduction of proliferation of stimulated non-Tregs: −2.2% (−8.7.1 to 77.7) vs. 32.7% (−0.4 to 77.9); *p* = 0.008].

Given that TNF-α and IFN-γ were shown to interfere with Treg function *in vitro* and that CD28^−^ Treg-like cells produced high levels of these cytokines (38, 39), we tested whether the suppressive capacity of CD28^−^ Treg-like cells was restored by the blockade of TNF-α or IFN-γ. The addition of neutralizing antibodies had no effect on the suppressive function of CD28^−^ Treg-like cells or CD28^+^ Tregs (Figures 5B,C).

### CD28^−^ Treg-Like Cells Are Prone to Apoptosis

Regulatory T-cells from CD28^−^ deficient mice have a pronounced proliferative/survival disadvantage (19). Therefore, we analyzed the proliferative capacity and apoptosis induction of CD28^−^ Treg-like cells. Upon stimulation with anti-CD3, we observed a lower rate of cell division of CD28^−^ Treg-like cells compared to CD28^+^ Tregs [53.1% CFSE^high^ (range 14.5–93.6) vs. 5.4% (0.9–30.6), respectively; *p* = 0.008; Figure 5D]. In addition, we performed Annexin V staining assays in order to analyze apoptosis induction this subset. In the CD28^−^ Treg-like subset, increased frequencies of early apoptotic [7% (range 2–25.7) vs. 1% (0.5–2.5); *p* = 0.005], necrotic [1.9% (0–9.2) vs. 0.2% (0.1–0.6); *p* = 0.011], and late apoptotic cells [17.1% (7.5–51.7) vs. 2.6% (1.3–8.4); *p* = 0.005] were observed upon stimulation with anti-CD3 compared to the CD28^+^ Treg population. Overall, more than 30% of CD28^−^ Treg-like cells were apoptotic or necrotic upon activation compared to only 5% of CD28^+^ Tregs (Figure 5E).

## Discussion

In the present study, we describe the novel CD4^+^ CD28^−^FoxP3^+^ T-cell subset. These cells reveal a phenotype compatible with senescent Tregs, have a reduced suppressive capacity compared to conventional CD4^+^ CD28^+^ FoxP3^+^ Tregs, show an increased production of pro- and anti-inflammatory cytokines, and display a high apoptosis rate. CD28^−^ Treg-like cells can be generated *in vitro* upon stimulation with TNF-α *in vitro*, and they are found in RA patients in peripheral blood and at sites of inflammation *in vivo*.

The increment of CD4^+^CD28^−^FoxP3^+^ T-cells in RA patients compared to HC was modest, albeit some individuals showed remarkable frequencies of this cell subset (with a maximum of 19.2% of all CD4^+^ T-cells). Given that Treg frequencies account for only ~5% of the total CD4^+^ T-cell population, even small changes in the composition of this subset might lead to major changes in the immune response, particularly in cases where the immune system is disturbed already, such as in RA. CD28^−^ Treg-like cells only weakly suppressed effector T-cells *in vitro* and released pro- as well as anti-inflammatory cytokines. Given that the ability to suppress effector cells is the most robust definition of Treg function, the question arises, whether CD28^−^ Treg-like cells still can be deemed as a regulatory subset. A major argument in favor of their regulatory origin is the expression of the transcription factor FoxP3. FoxP3 is essential for Treg development and function (27), and previous studies demonstrated that ectopic expression of FoxP3 in effector T-cells by retroviral gene transfer is sufficient to induce a Treg-like phenotype and function (40, 41). Besides, CD28^−^ Treg-like cells had strongly upregulated inhibitory surface molecules such as PD-1 and CTLA-4 and produced the immunosuppressive cytokine IL-10 (even more than CD28^+^ Tregs).

The most plausible alternative interpretation of our findings is that CD4^+^CD28^−^FoxP3^+^ T-cells are senescent non-Tregs that have transiently upregulated FoxP3 upon stimulation. CD4^+^CD28^−^FoxP3^+^ T-cells, however, were not only observed in RA patients with active disease but also in those in remission, and it is unlikely that CD4^+^CD28^−^ T-cells are continuously stimulated in patients where all other disease activity parameters are low. In addition, transient FoxP3 expression has been reported for naïve T-cells only, whereas memory and effector T-cells (which CD28^−^ T-cells belong to) seem not to upregulate this factor (42).

CD28^−^ Treg-like cells described in the present study are similar to Tregs identified in a mouse model with a conditional knock-out for CD28 (19). These animals suffer from severe autoimmunity due to defective Tregs, which are unable to suppress inflammation. Specifically, Tregs from the CD28 knockout mouse model yielded a similar phenotype to human CD28^−^ Treg-like cells such as the loss of CCR6 or the reduced expression of CD25, and an elevated production of IFN-γ. CCR6 is required for homing of Tregs to inflammatory sites (24), and Tregs of CD28^−^ deficient mice were virtually absent in inflamed skin lesions (43). In the synovial fluid of RA patients, CD28^−^ Treg-like cell levels were increased relative to circulation; however, this does not exclude a homing defect of CD28^−^ Treg-like cells because they could have been generated locally under the influence of pro-inflammatory cytokines such as TNF-α with subsequent efflux to periphery. Our *in vitro* experiments demonstrated that TNF-α downregulates CD28 on Tregs and TNF-α levels are known to be high in inflamed joints of RA patients (44). Nevertheless, TNF-α-induced CD28^−^ Tregs do not completely reflect CD28^−^ Treg-like cells described *in vivo* since expression levels of CD25 and CD127 were unaltered in *in vitro* stimulated cells. These differences are potentially caused by the length of stimulation and/or the presence of additional cytokines *in vivo*.

CD28^−^ Treg-like cells from RA patients had a clear survival disadvantage compared to CD28^+^ Tregs, which is again in line with the observations from the CD28 conditional knockout model (19). CD28^−^ Treg-like cells yielding a lower proliferative capacity plus a higher apoptosis rate are presumably functionally unable to halt autoimmune effector cells. Besides, CD28^−^ Treg-like cells from RA patients released several pro-inflammatory cytokines such as IFN-γ or TNF-α which might further promote the inflammatory process (38). Previous studies reported that treatment of RA patients with TNFα blockers restores Treg function (5, 45), whereas in our group, no association of anti-TNF therapy (or any other clinical variable except for age) with CD28^−^ Treg-like cell prevalence or function was found. This might be explained by the clinical heterogeneity of our cohort and the small sample size that lacked the power to elucidate the relation between different clinical phenotypes, therapies and CD28^−^ Treg-like cells. Larger studies are certainly needed to clarify this issue.

CD28^−^ Treg-like cells from RA patients expressed lower levels of CD25 compared to Tregs, which might have had an impact on their regulatory function. CD25 has been reported to operationally differentiate naturally present, autoimmune-preventive Tregs from other T-cells and therefore mediate immunosuppression (27). On the other hand, functional Treg subsets with a lower expression of CD25 have been reported, as well: a proportion of Tregs from aged mice, for example, showed decreased expression of CD25 (46, 47). These CD25^low^ Tregs occurred predominantly in the spleen (48) but had comparable functional properties to CD25^+^ Tregs. Similarly, a CD4^+^CD25^low^FoxP3^+^ Treg population has been observed in SLE patients, and given that SLE patients have a prematurely aged immune system with accumulation of CD28^−^ T-cells (49), it is conceivable that (at least part of) this CD4^+^ CD25^low^FoxP3^+^ Treg population was CD28^−^. A more recent study reported enhanced frequencies of CD25-FoxP3^+^ T-cells in RA patients and a negative correlation of this subset with disease activity (50). Unfortunately, no functional experiments were conducted with these cells thus not allowing to elucidate the overlaps and differences between these cell populations and clearly link CD4^+^ CD25^low^FoxP3^+^ Tregs with CD28^−^ Treg-like cells.

The most important limitation of this study is the uncertain identification of human Tregs by flow cytometry. No combination of surface molecules enables specific identification and isolation of Tregs. The transcription factors FoxP3 is still deemed as the most specific Treg marker; however, activated naive T-cells without suppressive function may transiently express this factor, as well. Second, RA is a heterogeneous disease with various clinical phenotypes. It is likely that the pathophysiology differs between various clinical subsets, and ideally, experiments would have been performed in different groups, stratified by their clinical phenotype. Third, we were unable to clarify whether CD28^−^ Treg-like cells occur before the clinical onset of RA (thus presumably contributing directly to the pathogenesis of the disease) or whether they occur later in course, secondary to chronic inflammation. Previous studies investigating healthy individuals with a genetic risk for RA concluded that early T-cell senescence, which might also involve the Treg lineage, is in part genetically determined and might well occur before the clinical onset of disease (51).

In conclusion, we describe a novel T-cell subset combining a regulatory phenotype with signs of T-cell senescence. This novel subset might represent a late developmental stage of Tregs (CD4^+^CD28^−^FoxP3^+^ T-cell). CD28^−^ Treg-like cells revealed a reduced suppression of effector cells, produced pro- and anti-inflammatory cytokines, and had a survival disadvantage compared to CD28^+^ Tregs. The CD28^−^ Treg-like cell population might thus contribute to the pathogenic immune response in RA.

## Author Contributions

JF, WG, MS, and CDe designed the research study. JF, AR, RH, AF, and CS conducted the experiments and acquired data. JF, CDu, WS, MS, and CDe analyzed data. WS and WG provided reagents. JF, MS, and CDe wrote the manuscript.

## Conflict of Interest Statement

The authors declare that the research was conducted in the absence of any commercial or financial relationships that could be construed as a potential conflict of interest.

## Supplementary Material

Figure S1 **CD28- Treg-like cells are not correlated with disease parameters in RA**. Graphs **(A)** correlation of CD28- Treg-like cells with age in HC (blue) and RA patients (red); **(B)** correlation of CD28- Treg-like cells with disease duration and **(C)** correlation of CD28- Treg-like cells with DAS28 in RA patients. Correlation between variables was evaluated by the Spearman´s rank correlation coefficient.

## References

[1] Fessler J Felber A Duftner C Dejaco C. Therapeutic potential of regulatory T cells in autoimmune disorders. BioDrugs (2013) 27:281–91. 10.1007/s40259-013-0026-5 23580095

[2] Lawson CA Brown AK Bejarano V Douglas SH Burgoyne CH Greenstein AS Early rheumatoid arthritis is associated with a deficit in the CD4+CD25high regulatory T cell population in peripheral blood. Rheumatology (Oxford) (2006) 45:1210–7. 10.1093/rheumatology/kel089 16571607

[3] van Amelsfort JM Jacobs KM Bijlsma JW Lafeber FP Taams LS. CD4(+)CD25(+) regulatory T cells in rheumatoid arthritis: differences in the presence, phenotype, and function between peripheral blood and synovial fluid. Arthritis Rheum (2004) 50:2775–85. 10.1002/art.20499 15457445

[4] Han GM O’Neil-Andersen NJ Zurier RB Lawrence DA. CD4+CD25high T cell numbers are enriched in the peripheral blood of patients with rheumatoid arthritis. Cell Immunol (2008) 253:92–101. 10.1016/j.cellimm.2008.05.007 18649874 PMC2585376

[5] Ehrenstein MR Evans JG Singh A Moore S Warnes G Isenberg DA Compromised function of regulatory T cells in rheumatoid arthritis and reversal by anti-TNFalpha therapy. J Exp Med (2004) 200:277–85. 10.1084/jem.20040165 15280421 PMC2211983

[6] Nie H Zheng Y Li R Guo TB He D Fang L Phosphorylation of FOXP3 controls regulatory T cell function and is inhibited by TNF-α in rheumatoid arthritis. Nat Med (2013) 19:322–8. 10.1038/nm.3085 23396208

[7] Walter GJ Fleskens V Frederiksen KS Rajasekhar M Menon B Gerwien JG Phenotypic, functional, and gene expression profiling of peripheral CD45RA+ and CD45RO+ CD4+CD25+CD127(low) treg cells in patients with chronic rheumatoid arthritis. Arthritis Rheumatol (2016) 68:103–16. 10.1002/art.39408 26314565 PMC4832388

[8] Wing K Ekmark A Karlsson H Rudin A Suri-Payer E. Characterization of human CD25+ CD4+ T cells in thymus, cord and adult blood. Immunology (2002) 106:190–9. 10.1046/j.1365-2567.2002.01412.x 12047748 PMC1782718

[9] Takahata Y Nomura A Takada H Ohga S Furuno K Hikino S CD25+CD4+ T cells in human cord blood: an immunoregulatory subset with naive phenotype and specific expression of forkhead box p3 (Foxp3) gene. Exp Hematol (2004) 32:622–9. 10.1016/j.exphem.2004.03.012 15246158

[10] Seddiki N Santner-Nanan B Tangye SG Alexander SI Solomon M Lee S Persistence of naive CD45RA+ regulatory T cells in adult life. Blood (2006) 107:2830–8. 10.1182/blood-2005-06-2403 16332974

[11] Steinmann GG Klaus B Muller-Hermelink HK. The involution of the ageing human thymic epithelium is independent of puberty. A morphometric study. Scand J Immunol (1985) 22:563–75. 10.1111/j.1365-3083.1985.tb01916.x 4081647

[12] Mackall CL Bare CV Granger LA Sharrow SO Titus JA Gress RE. Thymic-independent T cell regeneration occurs via antigen-driven expansion of peripheral T cells resulting in a repertoire that is limited in diversity and prone to skewing. J Immunol (1996) 156:4609–16. 8648103

[13] Valmori D Merlo A Souleimanian NE Hesdorffer CS Ayyoub M. A peripheral circulating compartment of natural naive CD4 Tregs. J Clin Invest (2005) 115:1953–62. 10.1172/JCI23963 16007258 PMC1159133

[14] Goronzy JJ Matteson EL Fulbright JW Warrington KJ Chang-Miller A Hunder GG Prognostic markers of radiographic progression in early rheumatoid arthritis. Arthritis Rheum (2004) 50:43–54. 10.1002/art.11445 14730598

[15] Warrington KJ Takemura S Goronzy JJ Weyand CM. CD4+, CD28- T cells in rheumatoid arthritis patients combine features of the innate and adaptive immune systems. Arthritis Rheum (2001) 44:13–20. 10.1002/1529-0131(200101)44:1<13::AID-ANR3>3.0.CO;2-6 11212151

[16] Solomon DH Karlson EW Rimm EB Cannuscio CC Mandl LA Manson JE Cardiovascular morbidity and mortality in women diagnosed with rheumatoid arthritis. Circulation (2003) 107:1303–7. 10.1161/01.CIR.0000054612.26458.B2 12628952

[17] Koetz K Bryl E Spickschen K O’Fallon WM Goronzy JJ Weyand CM. T cell homeostasis in patients with rheumatoid arthritis. Proc Natl Acad Sci U S A (2000) 97:9203–8. 10.1073/pnas.97.16.9203 10922071 PMC16846

[18] Zhang X Nakajima T Goronzy JJ Weyand CM. Tissue trafficking patterns of effector memory CD4+ T cells in rheumatoid arthritis. Arthritis Rheum (2005) 52:3839–49. 10.1002/art.21482 16329093

[19] Zhang R Huynh A Whitcher G Chang J Maltzman JS Turka LA. An obligate cell-intrinsic function for CD28 in Tregs. J Clin Invest (2013) 123:580–93. 10.1172/JCI65013 23281398 PMC3561819

[20] van der Linden S Valkenburg HA Cats A Evaluation of diagnostic criteria for ankylosing spondylitis. A proposal for modification of the New York criteria. Arthritis Rheum (1984) 27:361–8. 10.1002/art.1780270401 6231933

[21] Smolen JS Breedveld FC Schiff MH Kalden JR Emery P Eberl G A simplified disease activity index for rheumatoid arthritis for use in clinical practice. Rheumatology (Oxford) (2003) 42:244–57. 10.1093/rheumatology/keg072 12595618

[22] van Gestel AM Haagsma CJ van Riel PL. Validation of rheumatoid arthritis improvement criteria that include simplified joint counts. Arthritis Rheum (1998) 41:1845–50. 10.1002/1529-0131(199810)41:10<1845:AID-ART17>3.0.CO;2-K 9778226

[23] Hingorani R Monteiro J Furie R Chartash E Navarrete C Pergolizzi R Oligoclonality of V beta 3 TCR chains in the CD8+ T cell population of rheumatoid arthritis patients. J Immunol (1996) 156:852–8. 8543842

[24] Fessler J Raicht A Husic R Ficjan A Duftner C Schwinger W Premature senescence of T-cell subsets in axial spondyloarthritis. Ann Rheum Dis (2016) 75:748–54. 10.1136/annrheumdis-2014-206119 25688074 PMC4819616

[25] Yang J Fan H Hao J Ren Y Chen L Li G Amelioration of acute graft-versus-host disease by adoptive transfer of ex vivo expanded human cord blood CD4+CD25+ forkhead box protein 3+ regulatory T cells is associated with the polarization of Treg/Th17 balance in a mouse model. Transfusion (2012) 52:1333–47. 10.1111/j.1537-2995.2011.03448.x 22098312

[26] Shevach EM Regulatory/suppressor T cells in health and disease. Arthritis Rheum (2004) 50:2721–4. 10.1002/art.20500 15457438

[27] Sakaguchi S. Naturally arising Foxp3-expressing CD25+CD4+ regulatory T cells in immunological tolerance to self and non-self. Nat Immunol (2005) 6:345–52. 10.1038/ni1178 15785760

[28] Yamazaki T Yang XO Chung Y Fukunaga A Nurieva R Pappu B CCR6 regulates the migration of inflammatory and regulatory T cells. J Immunol (2008) 181:8391–401. 10.4049/jimmunol.181.12.8391 19050256 PMC2752441

[29] Sedelnikova OA Horikawa I Zimonjic DB Popescu NC Bonner WM Barrett JC. Senescing human cells and ageing mice accumulate DNA lesions with unrepairable double-strand breaks. Nat Cell Biol (2004) 6:168–70. 10.1038/ncb1095 14755273

[30] Naylor K Li G Vallejo AN Lee WW Koetz K Bryl E The influence of age on T cell generation and TCR diversity. J Immunol (2005) 174:7446–52. 10.4049/jimmunol.174.11.7446 15905594

[31] Dimri GP Lee X Basile G Acosta M Scott G Roskelley C A biomarker that identifies senescent human cells in culture and in aging skin in vivo. Proc Natl Acad Sci U S A (1995) 92:9363–7. 10.1073/pnas.92.20.9363 7568133 PMC40985

[32] Hohensinner PJ Goronzy JJ Weyand CM. Telomere dysfunction, autoimmunity and aging. Aging Dis (2011) 2:524–37. 22396899 PMC3295061

[33] Aletaha D Smolen J. The simplified disease activity index (SDAI) and the clinical disease activity index (CDAI): a review of their usefulness and validity in rheumatoid arthritis. Clin Exp Rheumatol (2005) 23:S100–8. 16273793

[34] Bryl E Vallejo AN Weyand CM Goronzy JJ. Down-regulation of CD28 expression by TNF-alpha. J Immunol (2001) 167:3231–8. 10.4049/jimmunol.167.6.3231 11544310

[35] Yamada H Kaibara N Okano S Maeda T Shuto T Nakashima Y Interleukin-15 selectively expands CD57+ CD28- CD4+ T cells, which are increased in active rheumatoid arthritis. Clin Immunol (2007) 124:328–35. 10.1016/j.clim.2007.06.001 17644042

[36] Murai M Turovskaya O Kim G Madan R Karp CL Cheroutre H Interleukin 10 acts on regulatory T cells to maintain expression of the transcription factor Foxp3 and suppressive function in mice with colitis. Nat Immunol (2009) 10:1178–84. 10.1038/ni.1791 19783988 PMC2898179

[37] Tchkonia T Zhu Y van Deursen J Campisi J Kirkland JL. Cellular senescence and the senescent secretory phenotype: therapeutic opportunities. J Clin Invest (2013) 123:966–72. 10.1172/JCI64098 23454759 PMC3582125

[38] Valencia X Stephens G Goldbach-Mansky R Wilson M Shevach EM Lipsky PE. TNF downmodulates the function of human CD4+CD25hi T-regulatory cells. Blood (2006) 108:253–61. 10.1182/blood-2005-11-4567 16537805 PMC1895836

[39] Hernandez AL Kitz A Wu C Lowther DE Rodriguez DM Vudattu N Sodium chloride inhibits the suppressive function of FOXP3+ regulatory T cells. J Clin Invest (2015) 125:4212–22. 10.1172/JCI81151 26524592 PMC4639983

[40] Bennett CL Christie J Ramsdell F Brunkow ME Ferguson PJ Whitesell L The immune dysregulation, polyendocrinopathy, enteropathy, X-linked syndrome (IPEX) is caused by mutations of FOXP3. Nat Genet (2001) 27:20–1. 10.1038/83713 11137993

[41] Fontenot JD Gavin MA Rudensky AY. Foxp3 programs the development and function of CD4+CD25+ regulatory T cells. Nat Immunol (2003) 4:330–6. 10.1038/ni904 12612578

[42] Tran DQ Ramsey H Shevach EM. Induction of FOXP3 expression in naive human CD4+FOXP3 T cells by T-cell receptor stimulation is transforming growth factor-beta dependent but does not confer a regulatory phenotype. Blood (2007) 110:2983–90. 10.1182/blood-2007-06-094656 17644734 PMC2018674

[43] Zhang R Borges CM Fan MY Harris JE Turka LA. Requirement for CD28 in effector regulatory T cell differentiation, CCR6 induction, and skin homing. J Immunol (2015) 195:4154–61. 10.4049/jimmunol.1500945 26408668 PMC4610862

[44] Saxne T Palladino MA Heinegård D Talal N Wollheim FA Detection of tumor necrosis factor alpha but not tumor necrosis factor beta in rheumatoid arthritis synovial fluid and serum. Arthritis Rheum (1988) 31:1041–5. 10.1002/art.1780310816 3136775

[45] McGovern JL Nguyen DX Notley CA Mauri C Isenberg DA Ehrenstein MR. Th17 cells are restrained by Treg cells via the inhibition of interleukin-6 in patients with rheumatoid arthritis responding to anti-tumor necrosis factor antibody therapy. Arthritis Rheum (2012) 64:3129–38. 10.1002/art.34565 22674488

[46] Chougnet CA Tripathi P Lages CS Raynor J Sholl A Fink P A major role for Bim in regulatory T cell homeostasis. J Immunol (2011) 186:156–63. 10.4049/jimmunol.1001505 21098226 PMC3066029

[47] Nishioka T Shimizu J Iida R Yamazaki S Sakaguchi S CD4+CD25+Foxp3+ T cells and CD4+CD25-Foxp3+ T cells in aged mice. J Immunol (2006) 176:6586–93. 10.4049/jimmunol.176.11.6586 16709816

[48] Lages CS Suffia I Velilla PA Huang B Warshaw G Hildeman DA Functional regulatory T cells accumulate in aged hosts and promote chronic infectious disease reactivation. J Immunol (2008) 181:1835–48. 10.4049/jimmunol.181.3.1835 18641321 PMC2587319

[49] Bonelli M Savitskaya A Steiner CW Rath E Smolen JS Scheinecker C. Phenotypic and functional analysis of CD4+ CD25- Foxp3+ T cells in patients with systemic lupus erythematosus. J Immunol (2009) 182:1689–95. 10.4049/jimmunol.182.3.1689 19155519

[50] de Paz B Prado C Alperi-López M Ballina-García FJ Rodriguez-Carrio J López P Effects of glucocorticoid treatment on CD25−FOXP3+ population and cytokine-producing cells in rheumatoid arthritis. Rheumatology (Oxford) (2012) 51:1198–207. 10.1093/rheumatology/kes039 22447883

[51] Schonland SO Lopez C Widmann T Zimmer J Bryl E Goronzy JJ Premature telomeric loss in rheumatoid arthritis is genetically determined and involves both myeloid and lymphoid cell lineages. Proc Natl Acad Sci U S A (2003) 100:13471–6. 10.1073/pnas.2233561100 14578453 PMC263838

