# Supplementary material for: Novel Senescent Regulatory T-Cell Subset with Impaired Suppressive Function in Rheumatoid Arthritis
Source: Front Immunol. 2017 Mar 20;8:300. doi: 10.3389/fimmu.2017.00300 (PMC5357868; doi:10.3389/fimmu.2017.00300)
Supplement: Supplementary file 1 [file Table_1.DOCX]

*Supplementary table 1:* Patients‘ characteristics:

|  | HC | RA | p-value |
| --- | --- | --- | --- |
| Number | 75 | 84 |  |
| Age [years] ^†^ | 57.3 (±10) | 59.7 (±10.7) | 0.120 |
| Female, n (%) | 49 (65.3) | 62 (73.8) | 0.247 |
| Disease duration [years] ^‡^ | n.a. | 7.5 (0.2-37.7) |  |
| Disease activity scores: | | | |
| SDAI^‡^ | n.d. | 5.2 (0.1-34) |  |
| DAS28^‡^ | n.d. | 2.5 (0-4.9) |  |
| Laboratory data: | | | |
| ESR (mm/1^st^h)^‡^ | n.d. | 12 (1-95) |  |
| CRP [mg/l]^‡^ | n.d. | 5 (0-78) |  |
| Current medication: | | | |
| Corticosteroids, n (%) | 0 | 17 (20.2) |  |
| Biologicals, n (%) |  |  |  |
| *anti-TNFα* | 0 | 28 (33.3) |  |
| *Tocilizumab* | 0 | 15 (17.9) |  |
| *Abatacept* | 0 | 13 (15.5) |  |
| *Rituximab* | 0 | 6 (7.1) |  |
| DMARDs, n (%) |  |  |  |
| *Methotrexate* | 0 | 37 (44) |  |
| *Leflunomide* | 0 | 12 (14.3) |  |
| *Sulfasalazine* | 0 | 2 (2.4) |  |
| *Other* | 0 | 1 (1.2) |  |
| NSAIDs, n (%) |  |  |  |
| *Regularly* | 0 | 8 (9.5) |  |
| *on demand* | 0 | 54 (64.3) |  |

^†^mean (±standard deviation); ^‡^median (range)

CRP, C-reactive protein [0-5 mg/l]; DAS28, Disease Activity Score 28; DMARD, disease-modifying anti-rheumatic drugs; ESR, erythrocyte sedimentation rate [0-30 mm/h]; HCs, healthy controls; n, number; n.a., not applicable; n.d., not determined; NSAID, non-steroidal anti-inflammatory drugs; RA, rheumatoid arthritis; sDAI, simplified disease activity index
