# Supplementary material for: Novel Senescent Regulatory T-Cell Subset with Impaired Suppressive Function in Rheumatoid Arthritis
Source: Front Immunol. 2017 Mar 20;8:300. doi: 10.3389/fimmu.2017.00300 (PMC5357868; doi:10.3389/fimmu.2017.00300)
Supplement: Supplementary file 2 [file Table_2.DOCX]

*Supplementary Table 2*: Spectratyping primer and probe sequences:

| BV1 | CAA CAG TTC CCT GAC TTG CAC |
| --- | --- |
| BV2 | TCA ACC ATG CAA GCC TGA CCT |
| BV3 | TCT AGA GAG AAG AAG GAG CGC |
| BV4 | CAT ATG AGA GTG GAT TTG TCA TT |
| BV5.1 | TTC AGT GAG ACA CAG AGA AAC |
| BV5.2 | CCT AAC TAT AGC TCT GAG CTG |
| BV6 | AGG CCT GAG GGA TCC GTC TC |
| BV7 | CTG AAT GCC CCA ACA GCT CTC |
| BV8 | TAC TTT AAC AAC AAC GTT CCG |
| BV9 | AAA TCT CCA GAC AAA GCT CAC |
| BV10 | CTC CAA AAA CTC ATC CTG TAC CTT |
| BV11 | ACA GTC TCC AGA ATA AGG ACG |
| BV12 | GAC AAA GGA GAA GTC TCA GAT |
| BV13.1 | GAC CAA GGA GAA GTC CCC AAT |
| BV13.2 | GTT GGT GAG GGT ACA ACT GCC |
| BV14 | GTC TCT CGA AAA GAG AAG AGG AAT |
| BV15 | GTC TCT CGA CAG GCA CAG GCT |
| BV16 | GAG TCT AAA CAG GAT GAG TCC |
| BV17 | CAC AGA TAG TAA ATG ACT TTC AG |
| BV18 | GAG TCA GGA ATG CCA AAG GAA |
| BV19 | TCC TCT CAC TGT GAC ATC GGC CA |
| BV20 | TCT GAG GTG CCC CAG AAT CTC |
| BV21 | GAT ATG AGA ATG AGG AAG CAG |
| BV22 | CAG AGA AGT CTG AAA TAT TCG A |
| BV23 | TCA TTT CGT TTT ATG AAA AGA TGC |
| BV24 | AAA GAT TTT AAC AAT GAA GCA GAC |
| BC-R | CTT CTG ATG GCT CAA ACA C |
