# Supplementary figures and images for: Novel Senescent Regulatory T-Cell Subset with Impaired Suppressive Function in Rheumatoid Arthritis
Source: Front Immunol. 2017 Mar 20;8:300. doi: 10.3389/fimmu.2017.00300 (PMC5357868; doi:10.3389/fimmu.2017.00300)

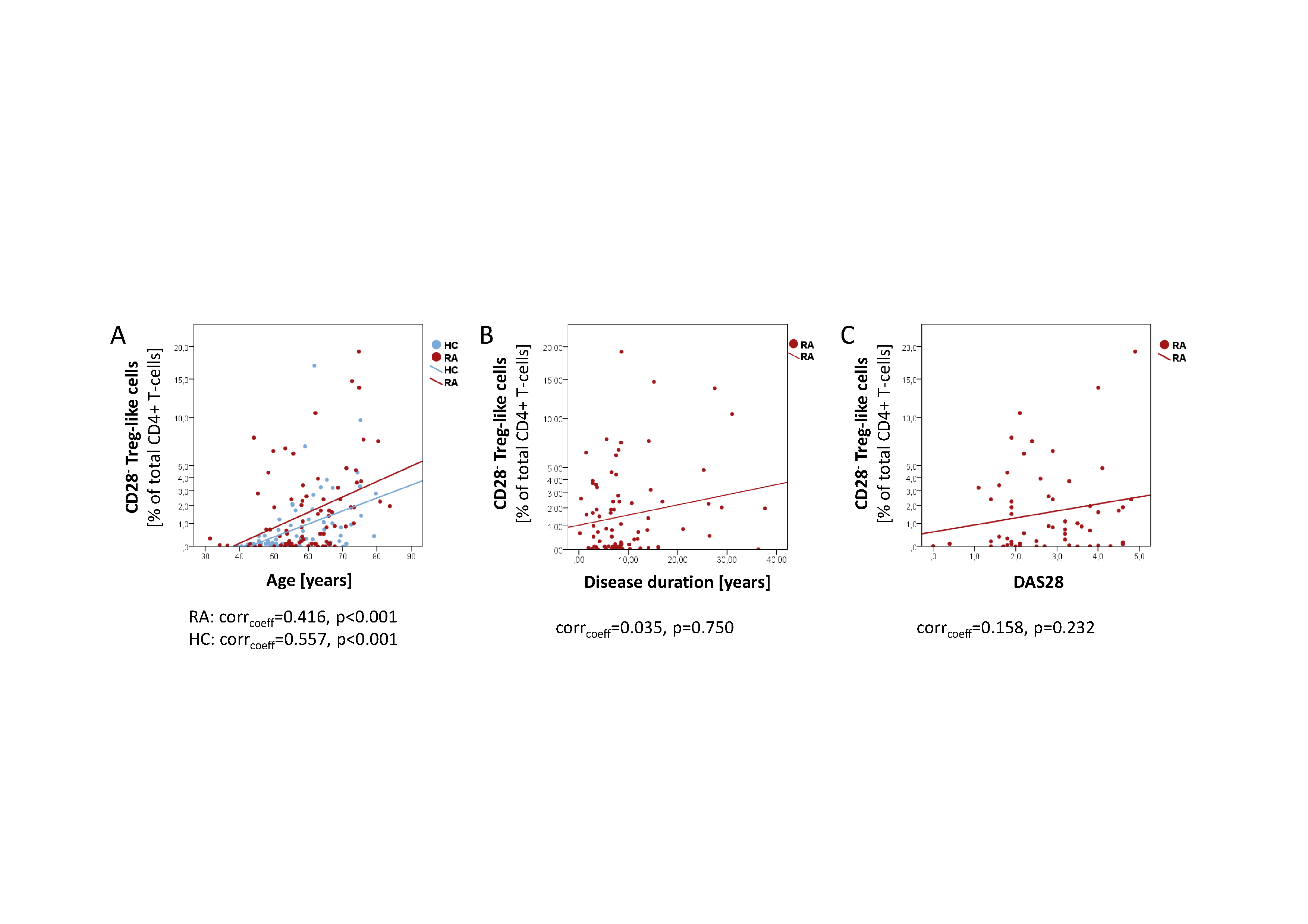

Supplement: Figure S1 — CD28- Treg-like cells are not correlated with disease parameters in RA. Graphs (A) correlation of CD28- Treg-like cells with age in HC (blue) and RA patients (red); (B) correlation of CD28- Treg-like cells with disease duration and (C) correlation of CD28- Treg-like cells with DAS28 in RA patients. Correlation between variables was evaluated by the Spearman´s rank correlation coefficient. [file Image_1.TIF]
